# Global Path Planning for Land–Air Amphibious Biomimetic Robot Based on Improved PPO

**DOI:** 10.3390/biomimetics11010025

**Published:** 2026-01-01

**Authors:** Weilai Jiang, Jingwei Liu, Wei Wang, Yaonan Wang

**Affiliations:** 1 The College of Artificial Intelligence and Robotics, Hunan University, Changsha 410082, China; jiangweilai@hnu.edu.cn (W.J.); yaonan@hnu.edu.cn (Y.W.); 2 Greater Bay Area Institute for Innovation, Hunan University, Guangzhou 511300, China; 3 The College of Architecture and Urban Planning, Hunan University, Changsha 410082, China; wangwei10731@hnu.edu.cn

**Keywords:** land–air amphibious biomimetic robot, path planning, reinforcement learning, PPO, GRU

## Abstract

To address the path planning challenges for land–air amphibious biomimetic robots in unstructured environments, this study proposes a global path planning algorithm based on an Improved Proximal Policy Optimization (IPPO) framework. Unlike traditional single-domain navigation, amphibious robots face significant kinematic discontinuities when switching between terrestrial and aerial modes. To mitigate this, we integrate a Gated Recurrent Unit (GRU) module into the policy network, enabling the agent to capture temporal dependencies and make smoother decisions during mode transitions. Furthermore, to enhance exploration efficiency and stability, we replace the standard Gaussian noise with Ornstein–Uhlenbeck (OU) noise, which generates temporally correlated actions aligned with the robot’s physical inertia. Additionally, a Multi-Head Self-Attention mechanism is introduced to the value network, allowing the agent to dynamically prioritize critical environmental features—such as narrow obstacles—over irrelevant background noise. The simulation results demonstrate that the proposed IPPO algorithm significantly outperforms standard PPO baselines, achieving higher convergence speed, improved path smoothness, and greater success rates in complex amphibious scenarios.

## 1. Introduction

Land–air amphibious biomimetic robots have garnered significant attention in recent years due to their unique ability to navigate complex, unstructured environments. By combining the high-speed mobility of aerial vehicles with the long-endurance capability of terrestrial robots, these platforms show immense potential in disaster rescue [1], military reconnaissance [2], and environmental monitoring [3]. Mimicking the versatile locomotion of biological organisms like birds [4] or insects, these robots can seamlessly transition between crawling on uneven terrain and aerial maneuvering to overcome obstacles. However, the dual-mode nature of these robots introduces severe challenges in motion control and path planning [5]. Unlike single-domain agents, amphibious robots must deal with distinct kinematic dynamics in different media and, more critically, ensure stability during the impulsive transition between ground and air modes. To provide a comprehensive context for our proposed solution, we review existing literature from three perspectives: classical path planning methods, reinforcement learning for single-domain robots, and emerging research on multi-modal platforms.

In the domain of classical path planning, methods are generally categorized based on the degree of environmental information available: global path planning, which utilizes complete known environmental data [6], and local path planning, which relies on real-time sensor data [7]. This paper focuses on planning an energy-efficient and path-optimal route within global maps, an area where traditional algorithms have established a strong foundation. For global search tasks, the A* algorithm remains a dominant baseline. Recent studies have optimized A* by improving heuristic functions and neighborhood search strategies (e.g., 24-neighborhood search) to significantly enhance search efficiency and path smoothness in static environments [8,9]. In high-dimensional complex environments, sampling-based methods have shown superiority; for instance, He et al. [10] developed an improved RRT*-Connect algorithm to successfully navigate high-DOF manipulators through multi-obstacle narrow passages. Regarding local planning and dynamic obstacle avoidance, P. et al. [11] proposed improved Artificial Potential Field (APF) methods to overcome the inherent “local minima” problem in real-time scenarios. Notably, specific to the domain of amphibious robots, Li et al. [12] recently adapted the Dynamic Window Approach (DWA) to handle the kinematic variations between water and land environments. However, despite these advancements, these traditional methods typically depend heavily on pre-built environmental models or deterministic kinematic constraints. They often struggle to adapt to the highly nonlinear dynamics and cross-domain mode switching (e.g., land-to-air) required by biomimetic robots, resulting in limited processing capabilities for such complex, unstructured scenarios.

To address the limitations of classical methods, Reinforcement Learning (RL)—particularly Deep Reinforcement Learning (DRL)—has been introduced to optimize behavior strategies through real-time trial-and-error interaction without relying on prior environmental knowledge [13]. In the ground domain, DRL has demonstrated robust performance in handling unstructured environments. For instance, Li et al. [14] utilized DRL to enable autonomous navigation of mobile robots in comprehensive unknown environments, significantly improving exploration efficiency. To address dynamic uncertainties and temporal dependencies, Zhang et al. [15] proposed an SAC-LSTM algorithm that integrates Long Short-Term Memory networks, allowing the agent to utilize historical information for rapid decision-making. Furthermore, Nie et al. [16] developed an enhanced Proximal Policy Optimization (PPO) algorithm with sample regularization and adaptive learning rates, which effectively improved path planning stability for Automated Guided Vehicles (AGVs). In the aerial domain, research has focused on 3D navigation and energy optimization. Fei et al. [17] applied DRL to achieve autonomous navigation and collision avoidance for UAVs in unknown environments. Addressing the complexity of three-dimensional spaces, Liu et al. [18] proposed an improved PPO algorithm for 3D path planning, enhancing convergence speed in complex scenarios. Additionally, considering the limited battery life of aerial platforms, Chen et al. [19] introduced an energy-efficient path planning method based on DRL. While recent advancements have begun to explore cross-domain applications—such as Cao et al. [20] applying SAC to amphibious UAVs (water-air) and Mondal et al. [21] investigating cooperative routing and planning for heterogeneous UAV-UGV systems using an attention-aware DRL framework—these works primarily focus on either fluid-air transitions or multi-agent collaboration. There remains a notable gap in unified global path planning for single-agent land–air biomimetic robots, specifically regarding the challenge of kinematic discontinuity during ground-air mode switching.

Emerging research on multi-modal platforms presents a different landscape. While recent hardware innovations have demonstrated the feasibility of such systems—as evidenced by Zhou et al.’s [22] comprehensive review, Mandralis et al.’s [23] mid-air morphing “ATMO” robot, and Zhang et al.’s [24] multimodal soft amphibious robot—motion planning strategies have largely lagged behind. Much of the existing literature focuses on the collaborative control of heterogeneous systems (e.g., UAV-UGV formations) [25], rather than single-agent autonomy. Research on single-agent global path planning is far less developed due to severe cross-domain kinematic constraints during media transitions, a challenge highlighted by Liang et al. [26]. Consequently, applying standard Deep Reinforcement Learning (DRL) frameworks to these complex environments often proves inadequate, manifesting as three critical operational failures specifically affecting amphibious robots. First, regarding temporal blindness, standard algorithms like PPO treat state-action pairs independently. This prevents the agent from retaining momentum information during ‘Kinematic Discontinuities’ (e.g., taking off from a slope), leading to abrupt thrust loss and instability. Second, regarding sparse rewards, unlike 2D rovers, amphibious robots operate in a vast 3D volumetric space where goal-directed signals are rare. This often traps the agent in ‘aimless loitering,’ causing it to miss narrow spatial corridors necessary for flight traversal. Finally, concerning exploration efficiency, generic strategies such as Gaussian noise generate uncorrelated, jerky control signals. For biomimetic robots with complex wheel-leg linkages, these high-frequency oscillations not only hinder convergence but also inflict mechanical stress, risking physical damage to actuators. Therefore, introducing a memory-capable mechanism and enhanced global perception into the decision-making process is crucial for maintaining trajectory smoothness and stability in hybrid environments.

To fill this gap, this paper proposes a global path planning algorithm based on an Improved Proximal Policy Optimization (IPPO) framework. Specifically designed for land–air amphibious biomimetic robots, this method ensures smooth and stable navigation across different media. The main contributions of this paper are summarized as follows:

(1) Amphibious Framework Design: We establish a global path planning framework specifically for land–air amphibious biomimetic robots, incorporating a comprehensive reward function that balances energy efficiency, safety, and mode-switching stability.

(2) Temporal Feature Extraction: A Gated Recurrent Unit (GRU) is integrated into the policy network. This module captures the temporal dependencies of the robot’s motion, effectively eliminating trajectory oscillations during the critical take-off and landing phases.

(3) Safe Exploration Mechanism: We replace the standard Gaussian noise with Ornstein-Uhlenbeck (OU) noise. This strategy generates temporally correlated exploration actions that align with the physical inertia of biomimetic joints, preventing mechanical damage from high-frequency control jitter.

(4) Enhanced Global Perception: A Multi-Head Self-Attention mechanism is introduced to the value network. This allows the agent to dynamically prioritize critical environmental features (e.g., narrow obstacles) over irrelevant background noise, significantly improving the success rate in cluttered environments.

## 2. Land–Air Amphibious Biomimetic Robot Platform

### 2.1. Land–Air Amphibious Biomimetic Robot Hardware Platform

**Figure 1:**
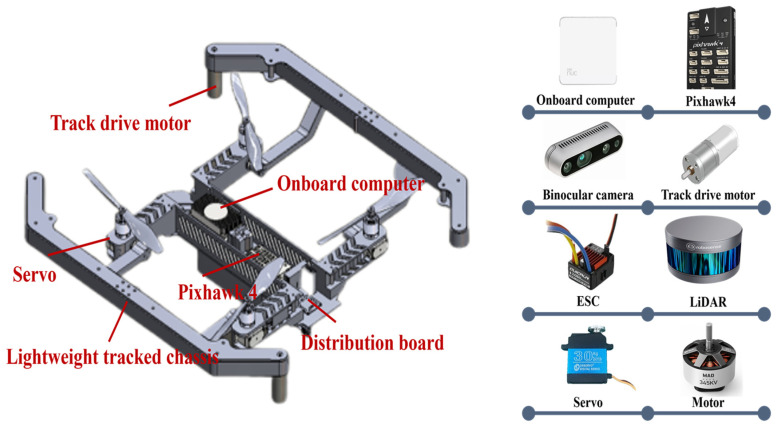
Appearance and hardware composition of the land–air amphibious biomimetic robot.

The land–air amphibious biomimetic robot has two modes: ground and flight. Its design needs to comprehensively consider the endurance in the form of drones and the passability in the form of unmanned vehicles. For this reason, this study adopts the deformable combination form of “multi-rotor drone + tracked unmanned vehicle”, as shown in Figure 1. Its hardware components include onboard computers, Pixhawk 4 flight control, binocular vision cameras, laser radars, etc.

**Figure 2:**
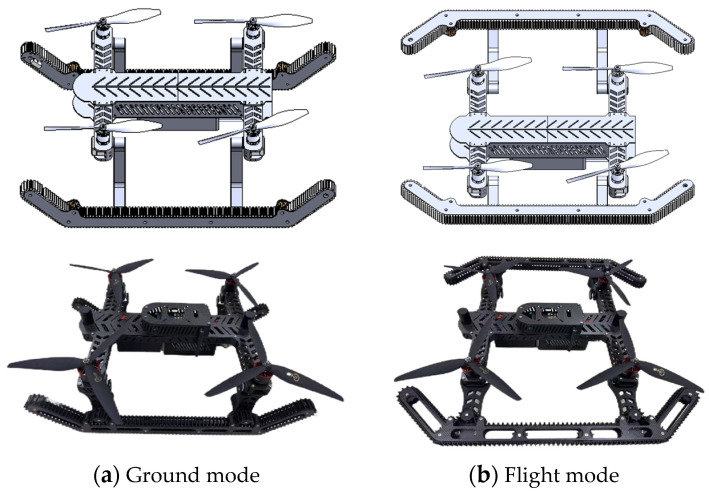
Mode of Land–air amphibious biomimetic robot.

Figure 2 is a mode diagram of the land–air amphibious biomimetic robot. When the robot is in ground mode, it uses a tracked driving device with stronger environmental adaptability. The flight mode is a four-rotor aircraft. The multi-rotor design allows the robot to take off and land anytime and anywhere, and has good maneuverability. When changing from the unmanned vehicle form to the drone form, the tracks on both sides of the robot can be folded upward through the foldable track arms to transform into a protective frame for the propellers, and the folding time is 2 s. In this study, the flight speed of the land–air amphibious biomimetic robot is set at 2 m/s, and the ground movement speed is set at 1 m/s. Since the flight energy consumption is much higher than the ground energy consumption, the ground energy consumption is set at 46.4 J/m and the flight energy consumption is set at 270.3 J/m.

### 2.2. Kinematic Consistency and Energy Analysis

To facilitate efficient reinforcement learning training, we adopt a simplified average energy model based on the Cost of Transport (CoT). While flight energy physically depends on complex aerodynamics (e.g., banking angle, acceleration) and ground energy on terrain interaction, modeling these micro-dynamics at the planning level introduces excessive computational overhead and sparse reward noise.

Therefore, we linearize the energy consumption as:

(1) $$E_{total}\approx C_{air}\cdot D_{air}+C_{groud}\cdot D_{groud}$$

where D represents the path length. The coefficients $C_{groud}=46.4$ J/m and $C_{air}=270.3$ J/m are derived from the empirical average power consumption of the robot at its nominal cruise velocities.

The justification for this simplification is threefold. First, regarding the macro-decision focus, the primary objective of the IPPO agent is to optimize high-level mode-switching strategies, where the significant magnitude disparity (≈1:6) between ground and air costs serves as the critical decision driver rather than minute fluctuations caused by instantaneous friction or drag. Second, in terms of computational efficiency, adopting a linear energy model significantly accelerates the reward calculation process throughout the millions of training steps required, thereby ensuring the overall feasibility of the algorithm. Finally, this approach enhances robustness; by employing conservative average values, we prevent the agent from exploiting simulation loopholes—such as unrealistic ‘gliding’ without energy cost—and guarantee that the planned paths remain valid and transferable under real-world disturbances.

## 3. The PPO Algorithm

Given the high-dimensional continuous action space defined by the dual-mode operation (flight and ground driving) described in Section 2, discrete control algorithms are insufficient. Therefore, we adopt the Proximal Policy Optimization (PPO) algorithm as our foundational framework. PPO is selected for its stability in continuous control tasks. However, as noted in our contributions, the standard “Vanilla PPO” lacks the temporal memory and noise filtration capabilities required for amphibious navigation. The mathematical formulation of the standard PPO is presented below, followed by our specific improvements.

**Figure 3:**
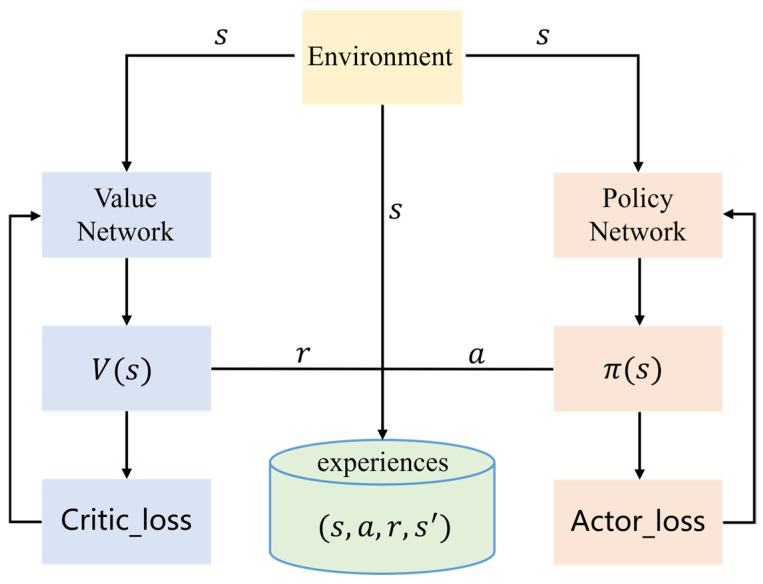
PPO algorithm framework diagram.

The PPO algorithm is an Actor-Critic based method, with its structure illustrated in Figure 3. The core architecture of this algorithm consists of two key components: the policy network (Actor) and the value network (Critic). Specifically, the policy network generates state-to-action mappings by outputting probability distributions over the action space, while the value network focuses on estimating the state-value function to compute advantage functions, thereby guiding policy optimization and enhancing training stability.

The PPO algorithm enhances the policy network’s update process through the introduction of importance sampling and advantage functions, thereby improving sample utilization efficiency. Importance sampling is a method that estimates the expected value of one distribution by sampling data from a known distribution and weighting the samples accordingly. Given a random variable *x* following probability distribution *p*(*x*), the expected value of function *f*(*x*) is calculated as follows:

(2) $$E_{x\sim p(x)}[f(x)]=\int f(x)\cdot p(x)dx$$

If sampling directly from *p*(*x*) becomes difficult, we may instead sample from an alternative distribution *q*(*x*). In this case, the expected value calculation formula becomes Equation (3):

(3) $$E_{x\sim p(x)}[f(x)]=\int f(x)\cdot \frac{p(x)}{q(x)}q(x)dx=E_{x\sim q(x)}[f(x)\cdot \frac{p(x)}{q(x)}]$$

**Figure 4:**
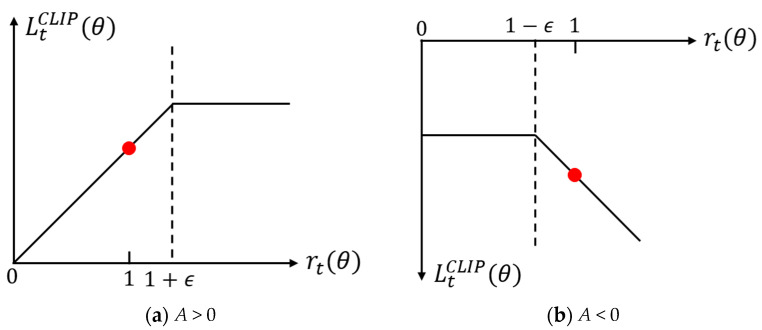
Objective function constraint range. The red dots represent examples where the probability ratio *r_t_*(*θ*) falls within the unclipped interval (i.e., the update is not clipped by *ε*), indicating that the standard gradient update is active.

In PPO, the goal of the policy network is to maximize the expected return of the new policy. However, since the new policy has not yet interacted with the environment and cannot directly sample data, it needs to rely on samples generated by the old policy. PPO uses importance sampling to update the new policy using sample data generated by the old policy. However, direct use of importance sampling may cause the policy update to be too large, affecting the stability of training. To this end, PPO designs an objective function that contains a clipping term, which constrains the policy update by limiting the range of variation in the ratio of the new and old policies, thereby avoiding the complex optimization process of the KL divergence constraint, significantly improving the computational efficiency and ease of implementation of the algorithm while ensuring performance. The main calculation formulas are shown in Equations (4) and (5):

(4) $$r_{t}(\theta )=\frac{\pi_{\theta }(a_{t}|s_{t})}{\pi_{\theta_{old}}(a_{t}|s_{t})}$$

(5) $$L_{t}^{CLIP}(\theta )={\hat{E}}_{t}[\min(r_{t}(\theta ){\hat{A}}_{t},clip(r_{t}(\theta ),1-\varepsilon ,1+\varepsilon ){\hat{A}}_{t})]$$

where $r_{t}(\theta )$ represents the probability ratio between the new and old policies for selecting action $a_{t}$ in state $s_{t}$, ${\hat{A}}_{t}$ represents the advantage function, which is usually Generalized Advantage Estimation(GAE), θ represents the parameters of the policy network, and ε is the clipping range hyperparameter, which is used to limit the range of $r_{t}(\theta )$, usually set to 0.1 or 0.2. When the advantage function is greater than 0, it indicates that the current action is better than the average level, and its selection probability should be increased. At the same time, the upper limit of the ratio of the new and old strategies should be constrained to prevent excessive updates. On the contrary, when the advantage function is less than 0, it indicates that the current action is poor, and its selection probability should be reduced, and the lower limit of the ratio of the new and old strategies should be constrained to avoid excessive updates. In this way, PPO limits the probability ratio $r_{t}(\theta )$ of the new and old strategies selecting action $a_{t}$ in state $s_{t}$ to the range of [1−ε,1+ε], thereby constraining the update amplitude of the policy network, preventing drastic fluctuations in policy performance, and ensuring the stability of training. The constraints of the objective function $L_{t}^{CLIP}$ are shown in Figure 4.

In PPO, the value network is updated by minimizing the mean squared error between the estimated state value $V_{\theta }(s_{t})$ and the target value $V_{t}^{target}$, as shown in Equation (6):

(6) $$L_{t}^{Value}(\theta )=E_{t}[{\left(V_{\theta }(s_{t})-V_{t}^{target}\right)}^{2}]$$

where θ represents the parameter of the value network.

To encourage exploration and prevent the policy from converging to local optima, PPO introduces an entropy bonus while employing a neural network architecture with shared parameters between the policy and value networks to improve training efficiency. The final loss function is given by Equation (7):

(7) $$L_{t}(\theta )={\hat{E}}_{t}[L_{t}^{CLIP}(\theta )-c_{1}L_{t}^{Value}(\theta )+c_{2}S[\pi_{\theta }](s_{t})]$$

where $c_{1}$ and $c_{2}$ are hyperparameters, which control the weights of the value loss term and the entropy reward term, respectively, and $S[\pi_{\theta }](s_{t})$ represents the entropy of strategy $\pi_{\theta }$ in state $s_{t}$.

## 4. Improved PPO Algorithm for Land–Air Environments

**Figure 5:**
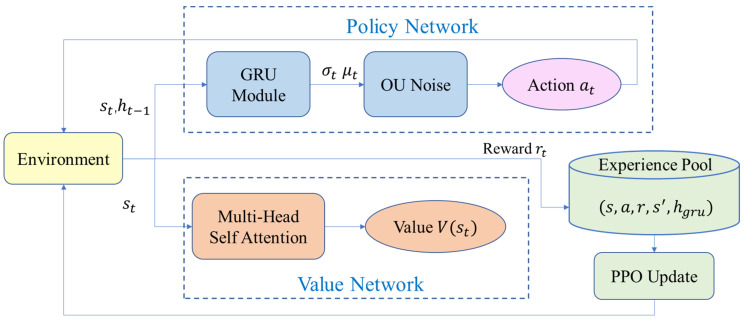
The overall framework of the proposed IPPO algorithm. The system comprises Environment Interaction, the Agent, and Learning modules. The Agent integrates a GRU for kinematic stability, Multi-Head Self-Attention for global perception, and OU noise for smooth exploration. Interactions are stored in the Replay Buffer to drive iterative PPO updates.

Although standard PPO provides a baseline for policy learning, its reliance on independent state processing and uncorrelated exploration noise proves inadequate for land–air amphibious robots. Specifically, this limitation exacerbates the ‘kinematic discontinuity’ problem highlighted in the Introduction, resulting in a loss of momentum information during mode transitions and generating mechanically harmful high-frequency control signals. To overcome these challenges, we present a novel IPPO algorithm that offers a holistic solution beyond generic hybrid methods. Our framework synergistically integrates three key enhancements: (1) a Gated Recurrent Unit (GRU) module to capture temporal dependencies for smooth trajectory generation; (2) Ornstein-Uhlenbeck (OU) noise to ensure temporally correlated, mechanically safe exploration; and (3) a Self-Attention mechanism to enhance global perception. These components work together to ensure seamless mode switching and robust path planning. The overall schematic of the proposed IPPO algorithm, detailing the interaction between these modules, is depicted in Figure 5 below.

### 4.1. Markov Decision Process of Land–Air Robot

#### 4.1.1. State Space

In order to comprehensively describe the real-time state of the land–air amphibious biomimetic robot, this subsection designs its state space, which overall includes the robot’s position, velocity, target information, and environment sensing data. The real-time state of the robot is firstly described by its position coordinates [x,y,z] and velocity information $\left[v_{x},v_{y},v_{z}\right]$, where the position coordinates represent the current position of the robot in the 3D space, and the velocity information reflects the robot’s motion state in the direction of each coordinate axis. In order to guide the robot to move towards the target point, the relative position of the target point and the robot [Δx,Δy,Δz] is introduced, which is calculated as:

(8) $$\Delta x=x_{p}-x_{c}\Delta y=y_{p}-y_{c}\Delta z=z_{p}-z_{c}$$

where $\left(x_{p},y_{p},z_{p}\right)$ represents the coordinates of the target point, $\left(x_{c},y_{c},z_{c}\right)$ represents the coordinates of the current position. In addition, the Euclidean distance D from the robot to the target point is introduced, and its calculation formula is:

(9) $$D=\sqrt{\Delta x^{2}+\Delta y^{2}+\Delta z^{2}}$$

This distance provides the robot with global positional information about the target point, facilitating path planning and navigation in complex environments. To enhance the robot’s environmental perception capabilities and enable autonomous obstacle avoidance and safe navigation, this study employs a 16-line 3D LiDAR as the primary sensor. Through feature extraction, the system obtains the nearest obstacle distances in 11 directions from each LiDAR data frame: front, left, right, front-left, front-right, front-up, front-down, front-upper-left, front-upper-right, front-lower-left, and front-lower-right, denoted, respectively, as:

(10) $$\left[s_{1},s_{2},s_{3},s_{4},s_{5},s_{6},s_{7},s_{8},s_{9},s_{10},s_{11}\right]$$

In summary, the state space $O_{t}$ of the land–air amphibious biomimetic robot at time *t* can be expressed as:

(11) $$O_{t}=\{x,y,z,v_{x},v_{y},v_{z},\Delta x,\Delta y,\Delta z,D,s_{1},\ldots ,s_{11}\}$$

#### 4.1.2. Action Space

In this paper, the action space of the land–air amphibious biomimetic robot is designed to be continuous, that is, the action space of the robot is described by the velocities $v_{x}$, $v_{y}$ and $v_{z}$ parallel to the *x*-axis, *y*-axis and *z*-axis of the global coordinate system. Therefore, the action space of the land–air amphibious biomimetic robot is expressed as:

(12) $$A=\{v_{x},v_{y},v_{z}\}$$

where the values of $v_{x}$, $v_{y}$ and $v_{z}$ are in the range [−1, 1].

#### 4.1.3. Reward Function

In reinforcement learning, the agent explores the environment by performing different actions, obtains corresponding reward values, and updates the strategy based on the reward, thereby gradually optimizing the behavior performance. Therefore, the reward function is the core element of completing the path planning task, and the rationality of its design will significantly affect the quality of path planning. However, the traditional reward function design usually only gives positive rewards when the agent reaches the target point, negative rewards when a collision or crossing the boundary occurs, and zero rewards in other cases. This reward function design has the problem of reward sparsity, which will cause the agent to receive delayed feedback when making decisions, thereby affecting strategy learning and may even cause the algorithm to not converge. Therefore, in this subsection, the reward function for path planning of land–air amphibious biomimetic robots is designed, which will be described in the following.

1. Distance reward. The distance reward is designed to encourage the land–air amphibious biomimetic robot to approach its target by measuring the change in Euclidean distance between the robot and the target point from the current timestep to the previous one, thereby rewarding progress made during path planning. Its mathematical expression is:

(13) $$r_{distance}=\eta (D_{t-1}-D_{t})$$

where $D_{t}$ and $D_{t-1}$ represent the Euclidean distances between the robot and the target point at the current timestep and the previous timestep, respectively, and η is the distance reward coefficient.

2. Altitude penalty. The land–air amphibious biomimetic robot has two modes: flight and ground travel, with the energy consumption of the flight mode being significantly higher than that of the ground mode. To reduce the robot’s energy consumption during flight and encourage ground travel, this paper introduces an altitude penalty. Its mathematical expression is:

(14) $$r_{height}=-\lambda \max(0,z_{t}-\delta )$$

where $z_{t}$ represents the robot’s current altitude, λ is the altitude penalty coefficient, and δ is the altitude threshold. When the robot’s altitude is below the threshold δ, no additional penalty is applied. However, if the altitude exceeds δ, a certain penalty is imposed.

3. Collision penalty. To ensure the safety of path planning for the land–air amphibious biomimetic robot in complex environments, this paper introduces a collision penalty to prevent collisions between the robot and obstacles. Its mathematical expression is:

(15) $$r_{collision}=-\mu L_{collision}$$

where $L_{collision}$ serves as the collision flag ($L_{collision}=1$ when a collision occurs, otherwise $L_{collision}=0$), and μ is the collision penalty coefficient. This penalty mechanism effectively guides the robot to proactively avoid obstacle zones during path planning, thereby enhancing both the safety and feasibility of the planned trajectory.

4. Time penalty. To encourage the land–air amphibious biomimetic robot to accomplish path planning efficiently, this paper introduces a time penalty, designed to prompt the robot to reach the target point quickly while minimizing unnecessary movements, thereby reducing mission execution time. Its mathematical expression is:

(16) $$r_{time}=-k$$

where *k* represents the time penalty coefficient. This penalty works by applying a small negative reward at each time step, guiding the robot to optimize path planning, improve execution efficiency, and reduce unnecessary movements, thereby achieving a more efficient path generation strategy.

5. Smooth reward. In continuous control tasks, excessively abrupt action variations may lead to system instability, increased energy consumption, and trajectory oscillations. To ensure the smoothness of robotic motions, this paper introduces a smooth reward. Its mathematical expression is:

(17) $$r_{smooth}=-\xi {\left\|a_{t}-a_{t-1}\right\|}^{2}$$

where $\left\|a_{t}-a_{t-1}\right\|$ represents the Euclidean distance variation between consecutive actions, and ξ is the smooth reward coefficient. This reward mechanism penalizes abrupt action changes, guiding the robot to generate smooth trajectories. It ensures motion stability, reduces unnecessary energy expenditure, and enhances control precision.

6. Obstacle traversal reward. To address scenarios where land–air amphibious biomimetic robots encounter impassable obstacles in ground mode within complex environments, this paper introduces an obstacle traversal reward to incentivize the robot to employ flight mode when necessary for obstacle clearance. Its mathematical expression is:

(18) $$r_{over}=\beta L_{obstacle}e^{-\frac{\left|z_{t}-h_{obs}\right|}{\sigma }}$$

where $L_{obstacle}$ is a cross-map obstacle detection flag, $h_{obs}$ represents the obstacle height, $z_{t}$ represents the robot’s current altitude, β is the reward coefficient, and σ is a tuning parameter. This reward reaches its maximum value when the robot’s altitude approaches the obstacle height, thereby effectively compensating for the altitude penalty during flight.

7. Terminal Reward. When the land–air amphibious biomimetic robot successfully reaches the target point, a significant positive reward is provided to incentivize task completion. Its mathematical expression is:

(19) $$r_{terminal}=R_{goal}$$

where $R_{goal}$ represents the terminal reward value, set to 800. This reward can effectively guide the robot toward optimal path planning by minimizing detours and ensuring timely arrival at the target, thereby significantly enhancing planning efficiency.

In summary, the expression of the integrated reward function designed in this paper is:

(20) $$R_{t}=r_{distance}+r_{height}+r_{collision}+r_{time}+r_{smooth}+r_{over}+r_{terminal}$$

However, simply aggregating these terms is insufficient; the efficacy of the algorithm hinges on the appropriate balancing of their respective coefficients. In terms of parameter configuration, rather than relying on fixed scalar values that limit generalization, we adopted a hierarchical tuning strategy to resolve potential reward interference and ensure robust convergence. Specifically, to prioritize safety, the collision penalty is set significantly higher than the maximum possible accumulated time costs, preventing the agent from intentionally crashing to terminate the episode early. Furthermore, to address the conflict between energy saving and obstacle traversal, the obstacle crossing reward is calibrated to outweigh the altitude penalty, ensuring the agent is motivated to switch to flight mode for overcoming barriers rather than being trapped in a ground-level local optimum. This relative scaling principle ensures that the global objective of safe and efficient navigation is maintained across varying environmental scales.

### 4.2. Improved Strategy Network with GRU

Land–air amphibious robots differ from standard UAVs or UGVs in that their current state is heavily influenced by previous momentum, especially during takeoff and landing phases. Feed-forward neural networks (as used in vanilla PPO) lack memory of these past states, often resulting in jerky control signals that can destabilize the robot. To address this, we embed a Gated Recurrent Unit (GRU) layer into the policy network. Unlike LSTM which involves complex gating mechanisms, the GRU offers a streamlined architecture that efficiently retains a “memory” of the robot’s kinematic history. This ensures smooth transitions between terrestrial and aerial modes and allows the agent to make decisions based on a trajectory of states rather than a single snapshot.

**Figure 6:**
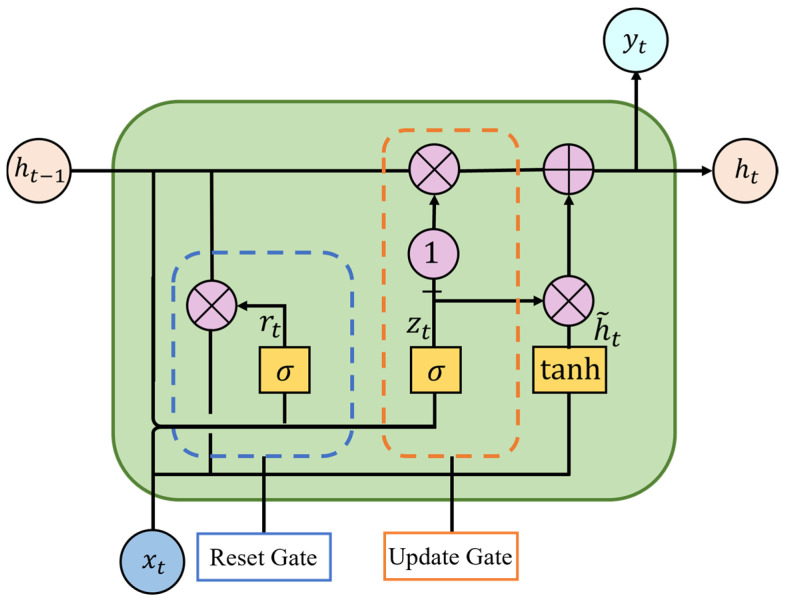
GRU network architecture.

The Gated Recurrent Unit (GRU) [27], an improved variant of Recurrent Neural Networks (RNN), effectively resolves the long-term dependency issues encountered by traditional RNNs in processing lengthy sequences through its innovative introduction of reset and update gates to regulate information flow. As shown in Figure 6, the GRU architecture primarily consists of these two gating mechanisms, where the reset gate controls the retention proportion of historical state information while the update gate determines how much historical information should be preserved versus new information incorporated in the current state.

As shown in Figure 6, the outputs of the reset gate and update gate are denoted as $r_{t}$ and $z_{t}$ , respectively, with their computational formulas given by Equations (21) and (22):

(21) $$r_{t}=\sigma (W_{r}\cdot [h_{t-1},x_{t}]+b_{r})$$

(22) $$z_{t}=\sigma (W_{z}\cdot [h_{t-1},x_{t}]+b_{z})$$

where $W_{r}$ and $W_{z}$ represent weight coefficients, $b_{r}$ and $b_{z}$ represent bias terms. Additionally, the GRU introduces the candidate hidden state ${\tilde{h}}_{t}$ at the current timestep to capture short-term dependencies, which is combined with the update gate $z_{t}$ to determine the final hidden state $h_{t}$ at the same timestep. The computational formulas for ${\tilde{h}}_{t}$ and $h_{t}$ are given by Equations (23) and (24):

(23) $${\tilde{h}}_{t}=\tanh(W_{\tilde{h}}[r_{t}\cdot h_{t-1},x_{t}]+b_{h})$$

(24) $$h_{t}=z_{t}\cdot {\tilde{h}}_{t}+(1-z_{t})\cdot h_{t-1}$$

**Figure 7:**
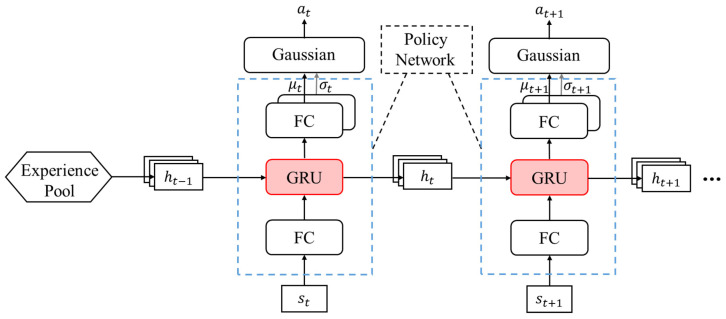
Policy Network Architecture of IPPO. This shows a three-layer network: feature extraction with ReLU, GRU for sequential modeling, and outputs for action mean and deviation. It highlights GRU’s role in optimizing continuous actions, backing the enhanced convergence speed in complex environments.

By leveraging the selective memory and state update mechanisms of GRU, incorporating it into the policy network of the PPO algorithm can effectively enhance the land–air amphibious biomimetic robot’s ability to extract historical state information, thereby optimizing the policy update process and significantly improving the convergence speed of the algorithm. The architecture of the proposed IPPO algorithm’s policy network is illustrated in Figure 7. The network consists of three layers. The first layer serves as a feature extraction module, where the input state information is passed through a fully connected layer with 128 hidden neurons for linear transformation, followed by a ReLU activation function to introduce nonlinearity and facilitate preliminary feature extraction. The second layer is a GRU, which performs sequential modeling on the extracted features, dynamically updating the hidden state to preserve historical dependencies. The third layer is the decision-making layer, in which the GRU output is fed into two separate fully connected layers, each containing 64 hidden neurons, to produce the mean and standard deviation of a Gaussian distribution that represents the continuous action space. Finally, the robot samples actions from this distribution and calculates the corresponding log-probability, which is then used for policy optimization and gradient-based updates.

### 4.3. OU Random Noise

Efficient exploration is critical for finding global optimal paths in complex environments. However, the independent Gaussian noise typically used in standard PPO generates uncorrelated, jittery action signals. For a biomimetic robot, such high-frequency oscillations can damage mechanical structures and cause unstable flight attitudes. To mitigate this, we employ the Ornstein-Uhlenbeck (OU) process to generate temporally correlated noise. OU noise models the velocity of a massive Brownian particle under friction, producing smoother exploration trajectories that align better with the physical inertia of the robot, thereby allowing for safe exploration without abrupt control commands. The differential equation form of OU noise is:

(25) $$dx_{t}=-\theta (x_{t}-\mu )dt+\sigma dW_{t}$$

where $x_{t}$ represents the state, $W_{t}$ represents the Wiener process, μ is the mean reversion rate, θ and σ are hyperparameters. Through discretization, the update rule for OU noise is formulated as:

(26) $$x_{t+\Delta t}=x_{t}-\theta (x_{t}-\mu )\Delta t+\sigma \sqrt{\Delta t}\varepsilon_{t}$$

where $\varepsilon_{t}$ is a random variable following the standard normal distribution.

**Figure 8:**
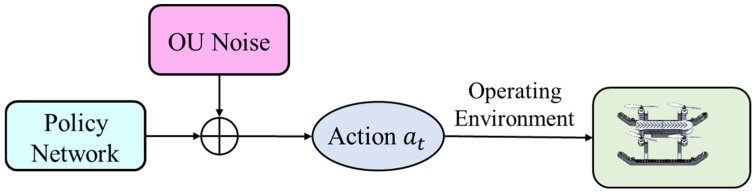
OU Noise Exploration Strategy.

The policy network architecture incorporating OU noise is illustrated in Figure 8. In this network, OU noise is additively applied to the action outputs, enabling the robot to generate smooth and coherent action sequences during exploration. This implementation not only enhances exploration efficiency but also significantly improves the stability and safety of path planning for the land–air amphibious biomimetic robot.

### 4.4. Improved Value Network with Self-Attention Mechanism

In complex environments filled with obstacles, traditional value networks often treat all sensory inputs equally, failing to distinguish between critical threats and irrelevant background noise. This limitation can lead to decision biases, especially when the robot needs to identify narrow passageways. To enhance global perception, we incorporate a Multi-Head Self-Attention mechanism into the value network. This mechanism dynamically computes correlations between different state features and adaptively assigns higher weights to critical environmental information. By focusing on global structural dependencies rather than just local sensor readings, the network significantly improves the accuracy of state-value estimation and the robustness of the planned path.

**Figure 9:**
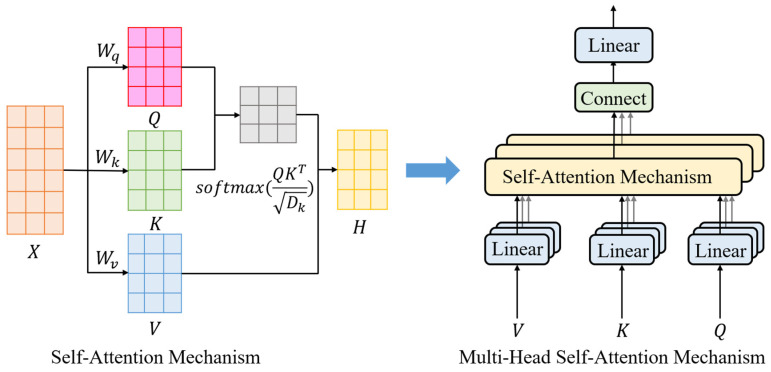
Computation process of multi-head self-attention. Diagram outlines linear transformations, attention weight calculation, weighted summation, and concatenation for multiple heads. It explains dynamic feature correlation, supporting better global perception in cluttered environments.

The multi-head self-attention mechanism [28], an advanced extension of self-attention, employs multiple parallel attention heads to enable simultaneous extraction of diverse representations from heterogeneous feature subspaces. This architecture significantly strengthens the network’s feature extraction capability and flexibility, demonstrating superior performance when processing complex sequential data. The computational process is illustrated in Figure 9.

Assume that there are *h* attention heads, each with an independent query, key, and value. Each attention head can extract features from the input sequence from different perspectives and learn the association information of the data in different subspaces. Since the parameters of each attention head are independent, they can capture diverse features, thereby enhancing the representation ability of the model. The specific calculation steps of the multi-head self-attention mechanism are as follows:

- Linear transformation: Assume that the input sequence is $X=[x_{1},\ldots ,x_{N}]\in R^{N\times D}$, where *N* is the length of the input sequence and *D* is the feature dimension of the input. For each attention head *i*, through different linear transformation matrices $W_{i}^{Q}$, $W_{i}^{k}$ and $W_{i}^{V}$, we obtain the query matrix $Q_{i}$, key matrix $K_{i}$ and value matrix. The calculation formula is shown in Formula (27):
  (27) $$Q_{i}=XW_{i}^{Q}\;K_{i}=XW_{i}^{K}\;V_{i}=XW_{i}^{V}$$
- Where $W_{i}^{Q}\in R^{D\times D_{q}}$, $W_{i}^{K}\in R^{D\times D_{k}}$, $W_{i}^{V}\in R^{D\times D_{v}}$.
- Calculate the attention weight: For each attention head, the dot product between the query matrix $Q_{i}$ and the transpose of the key matrix $K_{i}$ is calculated, and then divided by the scaling factor $\sqrt{D_{k}}$, and then normalized by the softmax function to obtain the attention weight matrix $A_{i}$. The calculation formula is shown in Formula (28):
  (28) $$A_{i}=\frac{Q_{i}K_{i}^{T}}{\sqrt{D_{k}}}$$
- Weighted summation: Multiply the attention weight matrix $A_{i}$ by the value matrix $V_{i}$ and perform a weighted summation to obtain the output $output_{i}$ of each head. The calculation formula is shown in Formula (29):
  (29) $$output_{i}=A_{i}V_{i}$$
- Concatenation and Fusion: The outputs of all h attention heads are concatenated and then transformed through a linear projection matrix $W^{o}$ to produce the final output. The calculation formula is shown in Formula (30):
  (30) $$output=Concat(output_{1},output_{2},\ldots ,output_{h})W^{o}$$

**Figure 10:**
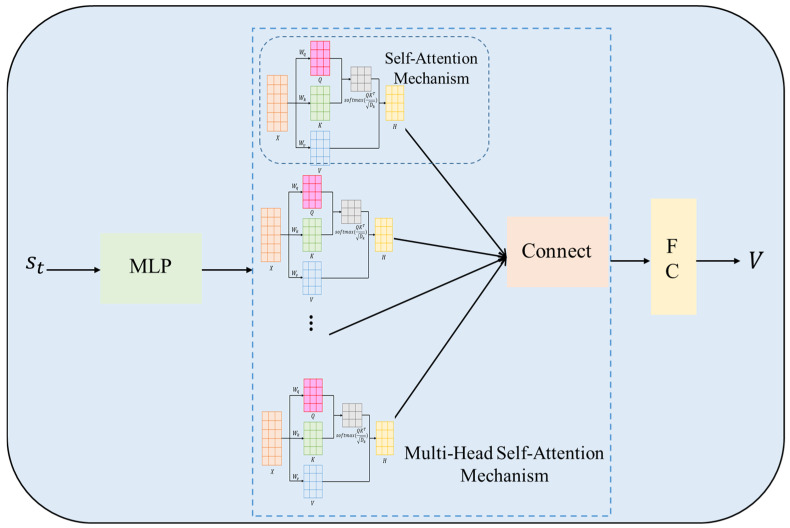
Value Network Architecture of IPPO. This depicts input through MLP with ReLU, multi-head self-attention, and final state value output. It shows how attention enhances state evaluation, supporting improved robustness and accuracy in path planning.

The value network structure of this paper, combined with the self-attention mechanism, is shown in Figure 10. First, the input state is subjected to preliminary feature extraction through a multilayer perceptron (MLP), and then nonlinearity is introduced through the ReLU activation function to enhance the model’s expressiveness. Next, the extracted features enter the multi-head self-attention mechanism layer, and the dependencies between different features are calculated in parallel through multiple attention heads to improve the network’s perception and robustness of global information. Finally, the features enhanced by the self-attention mechanism are input into the fully connected layer to output the state value. The improved value network can more accurately evaluate the value of the current state, accelerate convergence, and improve the overall performance and reliability of the algorithm.

To present the overall method framework more clearly, the algorithm pseudocode is shown in Algorithm 1:
**Algorithm 1:** Improved Proximal Policy Optimization (IPPO) for Amphibious Path Planning
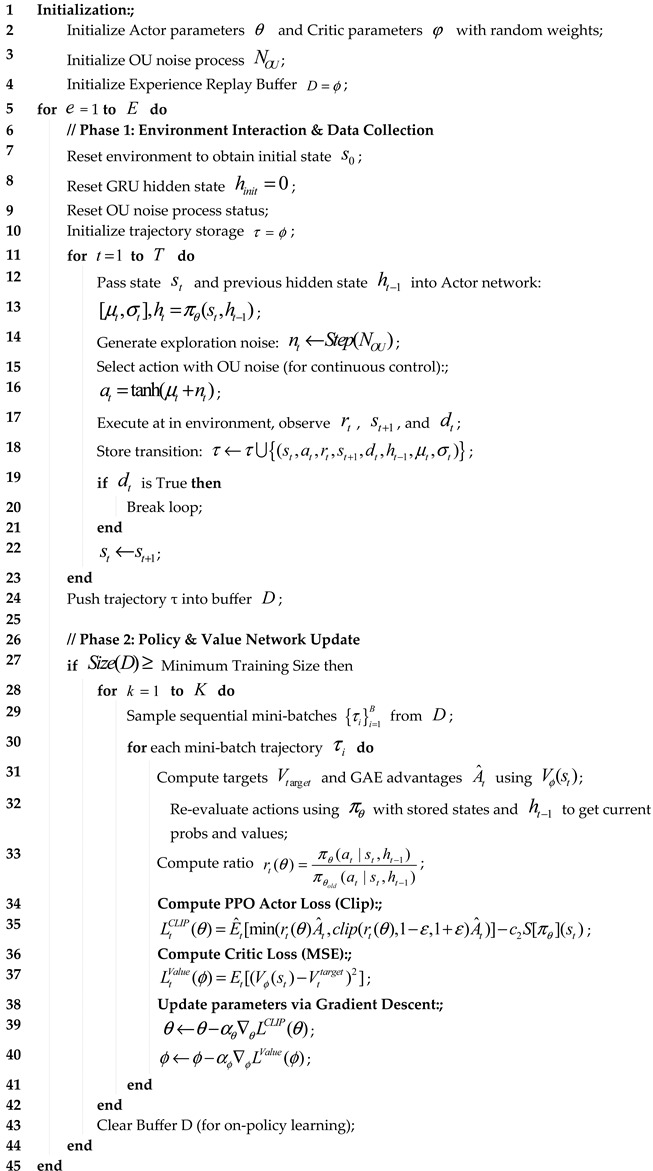


## 5. Experimental Results and Analysis

### 5.1. Environment and Parameter Configuration

**Figure 11:**
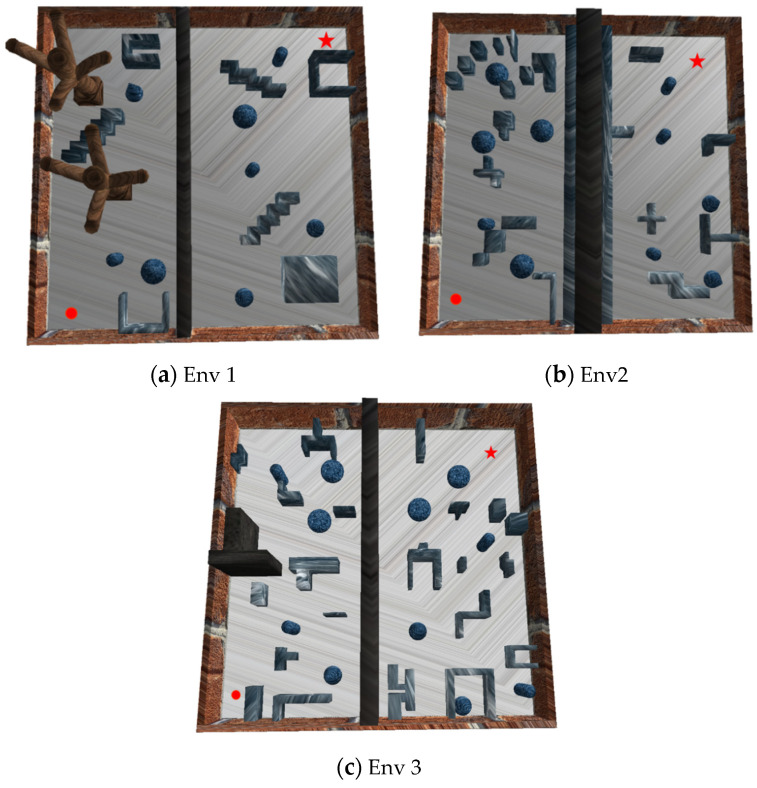
D simulation environment. The image displays three maps (Env 1–3) of 50 m × 50 m × 20 m with increasing obstacle complexity, marked start (red dot) and target (red star). It visualizes the testbed, supporting the algorithm’s generalization across varying environmental challenges.

The simulation platform is implemented using the Simulation Open Framework Architecture (SOFA) framework. We selected SOFA over standard rigid-body simulators (e.g., Gazebo) due to its superior capability in handling multi-physics interactions. This feature is particularly crucial for our biomimetic robot, as it allows for accurate modeling of the complex contact mechanics between the robot’s wheel-leg structure and deformable terrains, as well as the aerodynamic disturbances encountered during mode switching. Based on this high-fidelity platform, we developed three simulation environments with varying levels of complexity (Env 1 to Env 3) and encapsulated them as standard Gym interfaces to facilitate autonomous algorithm training. Detailed configurations of the specific geometric features, obstacle distribution, and computational resources of the simulation environments are summarized in Table 1. As illustrated in Figure 11, the spatial dimensions of all maps are standardized to 50 m × 50 m × 20 m, where the red dot and the red five-pointed star represent the starting point and the target destination, respectively. The environmental complexity increases progressively from Env 1 to Env 3, providing a rigorous testbed to verify the effectiveness and generalization capabilities of the proposed algorithm across diverse scenarios. The algorithm-specific hyperparameters were initialized based on established literature [16,18,27,28] and further fine-tuned through preliminary trials in the simulated amphibious environment. The specific parameter values are detailed in Table 2. To ensure fair comparison, all baseline algorithms were trained under identical environmental conditions.

To quantitatively evaluate the reliability of the proposed algorithm, we established clear termination criteria for each experimental episode. An episode is recorded as a Success when the robot successfully navigates from the starting point to the vicinity of the target destination without any collisions. Conversely, an episode is marked as a Failure if the robot collides with an obstacle or fails to reach the target within a reasonable operational timeframe. Based on these criteria, the Success Rate, which serves as a primary performance metric in our comparative analysis, is defined as the percentage of successful trials out of the total number of evaluation episodes.

### 5.2. Ablation Experiment

In order to verify the impact of the GRU network, OU random noise and self-attention mechanism of the IPPO improvements on the algorithm performance, this section conducts an ablation experiment in env 3. Env 3 has high complexity and intricate obstacle distribution, which can fully test the performance of the algorithm model. The algorithm settings for experimental comparison are shown in Table 3. By comparing the combinations of different improvements (“✓” means included, and “✗” means not included), the contribution of each improved part to the algorithm performance is analyzed.

**Figure 12:**
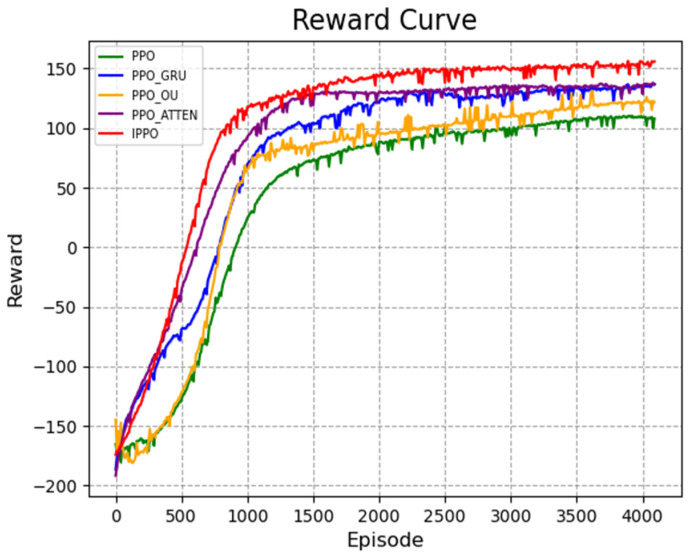
Average cumulative reward curve during training. The *x*-axis represents the number of training episodes, and the *y*-axis indicates the total dimensionless reward score. The curve is smoothed using a sliding window to highlight the overall convergence trend.

**Figure 13:**
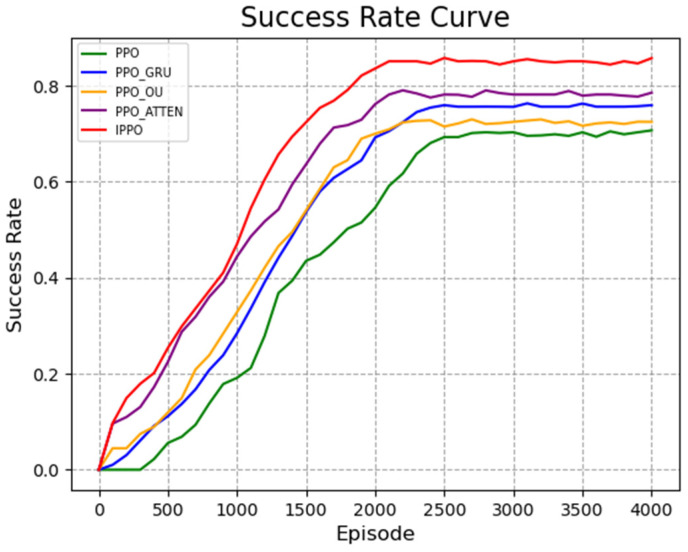
Success rate evaluation curve. The *y*-axis displays the success rate (ranging from 0 to 1), calculated as the ratio of successful arrivals to total attempts. The *x*-axis denotes the training progression in episodes.

The results of the ablation experiment are shown in Figure 12 and Figure 13. Figure 12 is the reward curve, and Figure 13 is the curve of calculating the success rate every 100 rounds. It can be seen from the figure that the traditional PPO algorithm has the slowest convergence speed, the final reward value is stable at about 110, and the success rate is only 70%, indicating that its strategy optimization efficiency is insufficient in complex environments.

The PPO_GRU algorithm can effectively capture the long-term dependencies in the sequence by introducing the GRU network. Its final reward value is stable at about 130, and the success rate reaches 75%, which is significantly improved compared with PPO. The PPO_OU algorithm improves the exploration ability by introducing OU noise. Its final reward value is about 120 and the success rate is 72%, which is slightly lower than PPO_GRU, but better than PPO. This shows that OU noise plays a certain positive role in the exploration process, but its effect is relatively limited. The PPO_ATTEN algorithm achieves a reasonable distribution of global state weights through the self-attention mechanism, significantly improving the directionality of strategy optimization. Its final reward value reaches about 130. The success rate reaches 70% within 1650 rounds and finally stabilizes at 80%, showing a high task completion efficiency. The IPPO algorithm performs best, with its final reward value stabilized at 150 and rapidly growing within 500 rounds, a success rate of up to 85%. In summary, the improved strategies GRU network, OU noise, and self-attention mechanism proposed in this paper have improved the performance of the algorithm to varying degrees, verifying its effectiveness.

### 5.3. Controlled Experiment

**Figure 14:**
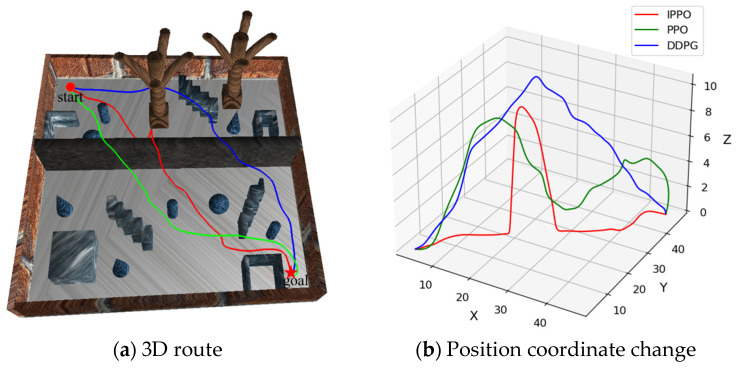
Path planning results in env 1. Trajectories of IPPO, PPO, and DDPG in a simple environment, with mode switches. IPPO’s path balances length and energy, supporting lower consumption via more ground travel.

**Figure 15:**
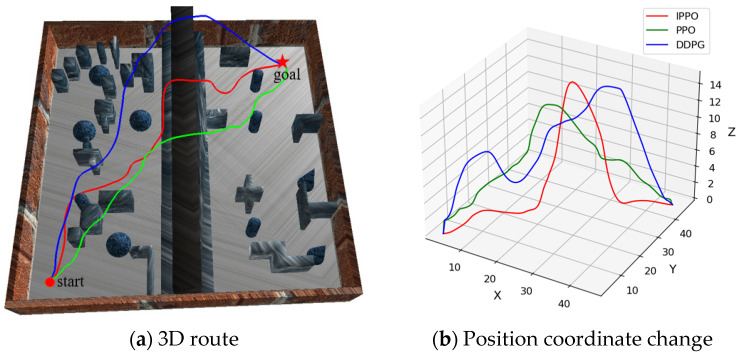
Path planning results in env 2. Paths in medium-complexity env, showing IPPO’s efficient mode use. It achieves shorter flight segments, backing claims of energy optimization and adaptability as complexity increases.

**Figure 16:**
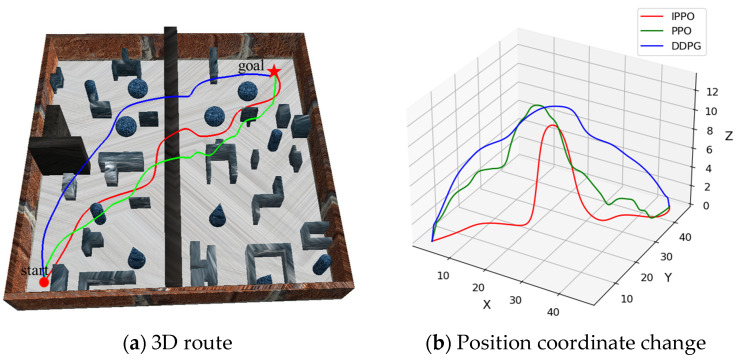
Path planning results in env 3. Trajectories in high-complexity env, with IPPO maintaining low energy despite obstacles. This illustrates robustness, supporting.

In the previous section, the effectiveness of the IPPO algorithm improvement strategy was verified through ablation experiments. In order to further verify the advantages of the IPPO algorithm proposed in this paper in the path planning of land–air amphibious biomimetic robots, this section designed and carried out comparative experiments to compare the performance of the IPPO algorithm with two traditional deep reinforcement learning algorithms PPO and DDPG. DDPG is an off-policy algorithm for continuous control that simultaneously learns a Q-function and a policy. These baselines were selected to represent different categories of RL algorithms: PPO as a classic on-policy method, and DDPG as representative off-policy methods. The experiments were conducted in env 1, env 2, and env 3, respectively. Each algorithm was run independently 25 times in each environment, and the average value of its performance indicators was calculated to ensure the stability and reliability of the results. By comparing and analyzing the performance of the three algorithms in different environments, the effectiveness, generalization, and robustness of the IPPO algorithm in path planning tasks are comprehensively evaluated. The path planning results in different environments are shown in Figure 14, Figure 15 and Figure 16, respectively. In these visualizations, the Ground Mode corresponds to low-altitude segments for energy saving, while the Flight Mode is characterized by vertical ascents specifically triggered for obstacle avoidance.

In this experiment, to simplify the complexity of the model, the amphibious robot is regarded as a point mass. Since the robot itself has a certain height, a height threshold is set in the experiment: when the flight height is higher than 0.2 m, it is considered to be in flight state, and when it is lower than 0.2 m, it is considered to be in ground driving state. At the same time, the flight energy consumption and ground driving energy consumption are set to 270.3 J/m and 46.4 J/m, respectively.

Table 4 Comparative experimental results under three environments records the performance indicators of each algorithm during the experiment, including average path length, average flight path length, average ground path length, and average energy consumption. It can be seen from Figure 14, Figure 15 and Figure 16 and Table 4 that the IPPO algorithm has significant advantages in the path planning task of land–air amphibious biomimetic robots, especially in terms of energy consumption optimization and environmental adaptability. With the increase in environmental complexity, IPPO can better balance path length and energy consumption, showing good robustness and generalization ability. In env 1 and env 2, the path length of the PPO algorithm is 76.041 m and 72.087 m, respectively, which is lower than that of the IPPO algorithm, but its energy consumption is much higher than that of IPPO. This implies that IPPO generates the most path-optimal trajectories, effectively minimizing redundant maneuvers and loitering in complex terrains. This efficiency is largely attributed to the temporal feature extraction capability of the GRU module, which smooths the control signals during mode transitions and reduces oscillatory behaviors. The path length of the DDPG algorithm in env 1 is lower than that of IPPO, and in env 2 is higher than that of IPPO, indicating that with the increase in environmental complexity, the stability of DDPG is poor. In addition, the energy consumption of DDPG in these two environments is higher than that of IPPO. In contrast, although the path length of the IPPO algorithm is slightly longer, it effectively reduces energy consumption by reducing the flight path and increasing ground travel. The energy consumption in env 1 and env 2 is 9.204 kJ and 12.661 kJ, respectively, which is significantly better than the other two algorithms. In env 3, as the complexity of the environment increases further, the IPPO algorithm still shows strong robustness and adaptability. Its path length is 75.181 m, close to PPO and DDPG, but the energy consumption is only 9.881 kJ, which is much lower than DDPG’s 18.778 kJ and PPO’s 17.416 kJ. In summary, the IPPO algorithm performs well in the path planning task of land–air amphibious biomimetic robots. It can effectively reduce energy consumption without significantly increasing the path length, giving full play to the advantages of land–air amphibious biomimetic robots. In addition, as the complexity of the environment increases, the IPPO algorithm remains stable and can flexibly respond to complex environmental changes, further demonstrating its superior generalization and reliability.

**Figure 17:**
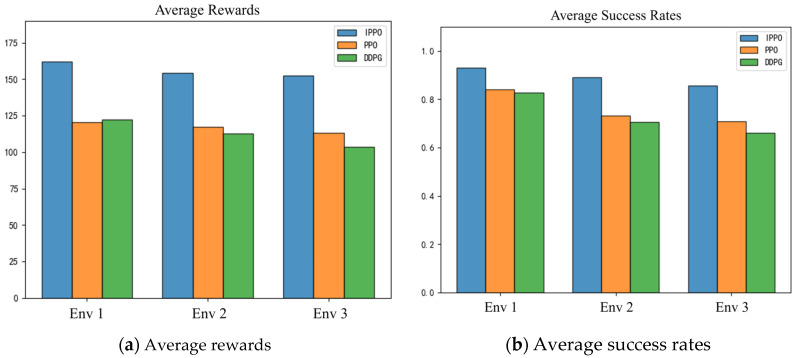
Visual comparison of average rewards and success rates. Bar charts for rewards and rates across envs 1–3. IPPO leads (e.g., 93% success in env 1), confirming stable high performance and environmental adaptability as complexity rises.

To further evaluate the performance of the IPPO algorithm, this section statistically analyzes the average rewards and success rates during the experiment. The results are shown in Table 5 and visualized as shown in Figure 17.

As can be seen from Table 5 and Figure 17, the average rewards and success rates of the IPPO algorithm in different environments are significantly better than those of DDPG and PPO, further verifying its superior performance in the path planning task of land -air amphibious robots. In terms of average reward, the average reward values of the IPPO algorithm in env 1, env 2, and env 3 are 162.2, 154.1, and 152.3, respectively, which are significantly higher than those of DDPG and PPO. In three environments, the average reward value of IPPO is about 25% higher than that of DDPG and PPO, indicating that the IPPO algorithm can effectively improve the quality of task completion. In terms of success rate, the IPPO algorithm also performed outstandingly, with success rates of 93.0%, 89.0% and 85.7% in env 1, env 2 and env 3, respectively, which are higher than DDPG and PPO. In addition, as can be seen from Figure 17, with the increase in environmental complexity, the average reward value and success rate of DDPG and PPO both show a downward trend, while the performance of the IPPO algorithm is relatively stable, which further verifies the environmental adaptability and generalization ability of the IPPO algorithm. In summary, the IPPO algorithm shows significant advantages in the path planning task of land–air amphibious biomimetic robots, can effectively improve the quality and success rate of task completion, and maintain strong adaptability and stability in complex environments, which fully demonstrates that the IPPO algorithm proposed in this paper has certain advantages.

It is important to note that the quantitative results presented in Table 5 are based on average performance metrics. While rigorous statistical tests (e.g., *t*-tests or ANOVA) were not conducted due to the computational constraints of large-scale reinforcement learning training, the magnitude of improvement observed in the proposed IPPO is substantial. For instance, the Success Rate surpasses the baseline DDPG by approximately 18%, and Energy Consumption is reduced by a significant margin. These large performance gaps suggest that the superiority of the IPPO framework is driven by the structural advantages of the GRU and Attention mechanisms rather than stochastic variance.

## 6. Conclusions

In this paper, we proposed a global path planning framework based on Improved Proximal Policy Optimization (IPPO), specifically tailored for land–air amphibious biomimetic robots to address the challenges of kinematic discontinuities and complex environmental interference. The proposed approach integrates a Gated Recurrent Unit (GRU), Ornstein–Uhlenbeck (OU) noise, and a Self-Attention mechanism into the standard PPO architecture to enhance robustness and adaptability.

The experimental results indicate that the integration of the GRU module successfully enables the agent to capture temporal dependencies, thereby reducing trajectory oscillations during mode switching and ensuring smoother transitions compared to memory-less baselines. Furthermore, the employment of OU noise facilitates temporally correlated exploration; unlike standard Gaussian noise, this strategy generates physically feasible control signals that mitigate the risk of mechanical damage associated with high-frequency jitter while improving convergence speed. Crucially, the Self-Attention mechanism effectively enhances global perception, allowing the agent to optimize the trade-off between trajectory length and energy efficiency. Quantitative comparisons based on average performance metrics demonstrate that the IPPO algorithm achieves a higher success rate and significantly lower energy consumption than standard PPO and DDPG baselines.

Despite these promising results, this study is subject to certain limitations, particularly the reliance on average metrics due to computational constraints and the exclusive use of a simulation environment. To address these challenges and advance the field, future research will focus on bridging the ‘Sim-to-Real’ gap by employing Domain Randomization techniques. This approach aims to train policies robust to real-world uncertainties, such as sensor noise and variable terrain friction, thereby facilitating the transfer of the IPPO algorithm to physical amphibious prototypes. Furthermore, we intend to extend the framework’s adaptability to dynamic environments by incorporating force-sensing feedback to mitigate unpredictable aerodynamic disturbances, such as wind gusts. Finally, integrating multi-modal sensor fusion, specifically by combining visual data from depth cameras with current state inputs, will be explored to enhance local perception fidelity, enabling more precise obstacle avoidance and autonomous landing site selection in complex, unstructured terrains.

## Figures and Tables

**Table 1:** Comprehensive summary of simulation environment configuration.

Category	Parameters	Value/Description
Simulation Platform	Physics Engine	SOFA
Global Environment	Map Dimensions	50 m × 50 m × 20m
	Obstacle Distribution	Spatially Dispersed, Locally Clustered
	Obstacle Density	3∼5%
	Obstacle Geometry	Cylinders: Radius 0.5 m–2.0 m Cuboids: Edge length 1.0 m–3.0 m Walls and irregular shapes
Computational Setup	Hardware	CPU: Intel Core i7-14700KF GPU: NVIDIA GeForce RTX 4060Ti RAM: 32 GB
	Software Stack	System: Ubuntu 20.04 Frameworks: Python 3.8, PyTorch 1.7.0.

**Table 2:** Hyperparameter settings of IPPO algorithm.

Parameters	Value
Training rounds	4100
Rewards discounts	0.995
Learning rate	0.00025
Clipping range	0.2
GAE factor	0.95
Batch_size	256
Maximum steps per episode	800
Optimizer	Adam
(η, λ, δ, μ)	(1.0, 5.0, 0.2, 100)
(k, ξ, β, σ)	(0.1, 0.1, 50, 0.5)

**Table 3:** Ablation experiment configuration.

Methods	GRU	OU	Self_Attention
PPO	✗	✗	✗
PPO_ATTEN	✗	✗	✓
PPO_OU	✗	✓	✗
PPO_GRU	✓	✗	✗
IPPO(Ours)	✓	✓	✓

**Table 4:** Comparative experimental results under three environments.

Env	Algorithm	Average Path Length/m	Average Flight Path Length/m	Average Ground Path Length/m	Average Energy Consumption/kJ
Env 1	DDPG	74.049	69.748	4.301	19.052
	PPO	76.041	72.536	3.505	19.769
	IPPO (Ours)	79.164	24.702	54.462	9.204
Env 2	DDPG	83.927	80.974	2.953	22.024
	PPO	72.087	69.675	2.412	18.945
	IPPO (Ours)	81.280	39.705	41.575	12.661
Env 3	DDPG	71.816	68.985	2.831	18.778
	PPO	73.086	62.639	10.447	17.416
	IPPO (Ours)	75.181	28.550	46.631	9.881

Table 4 details the quantitative performance of different algorithms across mixed environments. While multiple kinematic metrics are presented to provide a comprehensive view, the most critical performance differentiator is the ‘Energy Consumption’.

**Table 5:** Average rewards and success rates in different environments.

Env	Algorithm	Average Rewards	Average Success Rates
Env 1	DDPG	122.4	82.6
	PPO	120.6	84.0
	IPPO (Ours)	162.2	93.0
Env 2	DDPG	112.6	70.5
	PPO	117.5	73.3
	IPPO (Ours)	154.1	89.0
Env 3	DDPG	103.6	66.0
	PPO	113.2	70.8
	IPPO (Ours)	152.3	85.7

## Data Availability

The original contributions presented in the study are included in the article; further inquiries can be directed to the corresponding author.

## Abbreviations

The following abbreviations are used in this manuscript:

PPO	Proximal Policy Optimization
IPPO	Improved Proximal Policy Optimization
GRU	Gate Recurrent Unit
